# Physical and psychosocial challenges of people with gender dysphoria: a content analysis study

**DOI:** 10.1186/s12889-023-17537-z

**Published:** 2024-01-02

**Authors:** Zahra Ghiasi, Fatemeh Khazaei, Mohsen Khosravi, Nasrin Rezaee

**Affiliations:** 1 School of Medicine, Zahedan University of Medical Sciences, Zahedan, Iran; 2 Zahedan University of Medical Sciences, Zahedan, Iran; 3 Department of Psychiatry, Zahedan University of Medical Sciences, Zahedan, Iran; 4 Community Nursing Research Center, Zahedan University of Medical Sciences, Zahedan, Iran

**Keywords:** Psychosocial challenges, Gender dysphoria, Qualitative study

## Abstract

**Background:**

The mismatch between the gender experienced by a person and the gender attributed to him/her leads to gender dysphoria. It seems that people’s perception of gender dysphoria is affected by individual, cultural, and sociological factors and these factors affect different aspects of their biological, psychological, and social health. To this end, this qualitative study aimed to identify the physical, psychological, and social challenges of people with gender dysphoria referring to the Department of forensic medicine in Iran.

**Methods:**

This qualitative study was conducted using conventional content analysis on 9 individuals who were selected through purposive sampling. A total of 16 interviews were conducted with 9 participants. Each interview lasted 60–90 min. The participants’ gender dysphoria was confirmed by the Department of forensic medicine. The data were collected through face-to-face semi-structured interviews with the participants.

**Results:**

The data revealed 3 main categories and 10 subcategories. The main categories were living in agony, confusion, and social concerns. The subcategories were annoying physical characteristics, mental suffering, disturbing sexual changes, concerns about public reaction, helplessness, surrender, the final solution, retreating to isolation, stressful family conditions, and lack of public recognition.

**Conclusion:**

The findings showed that people with gender dysphoria suffer from some problems including living in agony, confusion, and social concerns. Each of these problems is associated with several challenges. It seems that most of the challenges faced by people with gender dysphoria are caused by unawareness of their conditions by the family and the public, which in turn is caused by the failure of related organizations and experts in this field to provide adequate information about the conditions of these people. Thus, the findings of the present study can have some implications for resolving the challenges faced by people with gender dysphoria.

## Background

Gender dysphoria (GD) refers to a person’s dissatisfaction with the sex assigned at birth. According to the diagnostic criteria, gender dysphoria refers to cases such as the mismatch between the gender experienced and the secondary or primary sexual characteristics of the person, a strong preference to get rid of the secondary or primary sexual characteristics, and a strong desire to have another gender [1]. Gender dysphoria is often associated with a person’s dissatisfaction with their physical appearance. An unfavorable physical appearance may lead to an unclear perception of gender identity. The mismatch between the gender experienced by an individual and the gender attributed to them in the community leads to the individual’s dissatisfaction [2]. Gender identity affects all aspects of people’s lives. People with gender dysphoria experience some degree of discrimination due to gender experiences that are not in line with the normal values of society [3]. Some of the people who cannot stand public judgments suffer many traumatic experiences in their daily life and as a result, they may hide their feelings about their gender identity to the extent that they even prefer to live in a different place and hide their past after sex reassignment surgery [4].

Gender dysphoria usually develops in early childhood and can remain stable for years until gender changes. Early diagnosis and measures during childhood and adolescence can reduce the probability of comorbidities and improve the quality of life of the affected person [5]. Studies conducted in Turkey, the Netherlands, and Iran show that gender reassignment surgery has a positive effect on the quality of life, family support, and interpersonal relationships of people with gender dysphoria [4, 6, 7]. A review study showed that people with gender dysphoria have a high risk of suffering from mental illnesses [8]. A majority of people with gender dysphoria may undergo stressful conditions due to gender identity conflict and discomfort with their biological gender [9]. A study in the United States showed that depression, anxiety, and suicidal thoughts are more prevalent among GD sufferers than in the general population [8]. Another study in the United States showed that gender reassignment improves the health, self-confidence, and quality of life of people with GD [9]. A study by Yıldızhan et al. (2018) in Turkey compared the lifestyle, social relationships, and quality of life of people with GD who had or had not performed gender reassignment surgery. The results showed people who had performed gender reassignment surgery had reduced anxiety related to gender discrimination, but increased anxiety related to disclosure of their past compared to the group who did not perform this surgery [4].

It seems that people’s perception of gender dysphoria is affected by individual, cultural, and sociological factors, and these factors affect various aspects of their biological, psychological, and social health. In Iran, gender reassignment and surgery are simply not possible for GD patients. In Iran, changing one’s legal gender can be a difficult and complex process. This is because the country’s legal system is based on Islamic law, which has specific rules and regulations regarding gender and related issues. Additionally, certain cultural and social attitudes toward gender can make it challenging for individuals to change their gender identity [10]. Thus, to better understand the challenges faced by these people, it is necessary to analyze their experiences and statements in a real context. Qualitative research helps the researcher to identify the challenges ahead by entering the inner world of these people and exploring their perspectives.

Sistan and Baluchistan province is located in the southeast of Iran. In this province, due to religious and cultural beliefs, it is difficult to talk about gender dissatisfaction, because it is Guilt and taboo. In addition, no institution provide support for GD individuals. However, no qualitative study has addressed the physical, psychological, and social challenges faced by people with gender dysphoria in the cultural and social context of Sistan and Baluchistan Province. Research findings in this field will help therapists to provide effective solutions for people with gender dysphoria to improve their health.

## Methods

### Aim

This study sought to identify the physical, psychological, and social challenges faced by people with gender dysphoria referring to the department of forensic medicine in Zahedan, Iran.

### Study design and setting

This qualitative study was conducted using conventional content analysis to explore the physical, psychological, and social challenges faced by people with gender dysphoria referring to the Department of forensic medicine in Zahedan. The conventional content analysis approach is a flexible method for analyzing textual data. This approach is used when there is not enough knowledge about a phenomenon. Using an inductive approach, conventional content analysis focuses on the creation and development of categories and the interpretation of written or spoken content [11].

### Participants

The participants in this study were selected through purposive sampling from people whose gender dysphoria was confirmed by the Department of forensic medicine and who were willing to participate in the study.

### Data collection

The data in this study were collected through face-to-face semi-structured interviews from June to late November 2022. The transcripts of the interviews were analyzed using conventional content analysis. A total of 16 interviews were conducted with 9 participants. Each interview lasted 60–90 min. The interviews were conducted in the forensic medicine department in a private room where the participants feel comfortable. Examples of the questions asked in the interview were as follows; How do/did you feel about your gender? How did you deal with it? How did you talk about it with your parents? How did your parents react to it? (Could you describe your memories?) Could you talk about your situation in the community (outside home/school/sports/extracurricular activities/workplace)? (Could you give some examples and describe your memories?) What irregular experience did you experience in the community? How do you interact with the people around you (family members, friends) and vice versa? How is their relationship with you? And what solutions do you think will help you to reduce your problems? Moreover, probing questions (e.g., what do you mean? Could you please give an example) were asked for further clarification. The interviews continued until data saturation i.e., when no new information was obtained from additional interviews. The participants’ interviews were recorded using a voice recorder and then transcribed into an MS Word file for subsequent analysis.

### Data analysis

Data analysis was done at the same time as data collection.To analyze the data, the texts of the interviews were read several times and then the primary codes were extracted. The extracted codes with similar meanings merged into more general clusters. The extracted clusters were revised and placed into some categories and subcategories around a core category [12].

In qualitative research, the rigor of the data is established through four criteria of dependability, credibility, transferability, and confirmability [13]. In this study, the researcher was engaged with the research procedure for 2 years. The rigor of the data was established through continuous reviews of the data i.e., transcribing the interviews, reviewing the data to extract the main categories, and revising the data through the member and peer checking. The dependability of the data was confirmed through the prolonged engagement with the data in all stages of the study.

## Results

Table 1 shows the participants’ demographic characteristics. The analysis of the data revealed 3 main categories and 10 subcategories. The main categories were living in agony, confusion, and social concerns. The subcategories were annoying physical characteristics, mental suffering, disturbing sexual changes, concerns about public reaction, helplessness, surrender, the final solution, retreating to isolation, stressful family conditions, and lack of public recognition as shown in Table 2.

**Table 1:** The participants’ demographic characteristics

Participant code	Age	Education	Occupation	Marital status	Living conditions	Initial gender	Reassigned gender	The age of understanding gender dysphoria by participants	Gender reassignment permit	The period under the supervision of the forensic medicine department
1*	17	6th grade	Student	Single	Supported by the welfare department	Female	Male	11 (year)	No	8 months
2*	16	6th grade	Student	Single	Supported by the welfare department	Female	Male	11 (year)	No	8 months
3*	35	High school diploma	Unemployed	Single	Living with a partner	Male	Female	5 (year)	No	6 months
4*	20	College student	College student	Single	Living with parents	Female	Male	5 (year)	No	12 months
5*	24	College student	College student	Single	Living with parents	Male	Female	9 (year)	No	15 months
6*	43	High school diploma	Painter	Single	Living with parents	Female	Male	5 (year)	Gender affirmation	36 months
7	24	Bachelor’s degree	Unemployed	Single	Living with parents	Female	Male	4 (year)	Gender affirmation	18 months
8	23	College student	College student	Single	Living with parents	Female	Male	4 (year)	No	1 month
9*	23	Bachelor’s degree	Unemployed	Single	Living with parents	Male	Female	5 (year)	No	6 months

* The participant attended an additional interview

**Table 2:** The main categories and subcategories

Themes	Subcategories	Main categories
Suffering from the male appearance (#9) Hiding feminine characteristics (#2) Dislike of female characteristics (#4) Hatred of one’s feminine appearance (#1)	Annoying physical characteristics	Living in agony
Deprived of the simplest blessing (gender identity) (#1) Living in hell (#6) Endless harassment from acquaintances (#6) Mental health stigma (#7) Being ridiculed (#9)	Mental suffering	
Concerns about life after the sexual reassignment surgery (#3) Concerns about post-marital life (#5) Concerns about one’s appearance after the surgery (#7) Concerns about fertility after the surgery (#9)	Disturbing sexual changes	
Concerns about public curiosity about one’s previous sexual identity (#8) Fear of public rejection after the surgery (#1) Concerns about public failure to recognize the new identity (#1) Fear of the disclosure of the previous identity (#1)	Concerns about public reaction	
Confusion about sexual identity (#5) Identity problems (#5) Complaining about God (#6) Instability (#5)	Helplessness	Confusion
Surrender to others’ surprise (#5) Wearing clothes according to local norms (#5) Family conflict (#5) Futile attempts to keep the apparent identity (#9)	Surrender	
Preparing for independence or rejection after reassignment surgery (#3) Suicidal thoughts in times of helplessness (#4) Preference to have a specific gender identity (#6) Migration to find work (#6)	The final solution	
Feeling safe in being locked up (#8) Reduction of interactions due to appearance (#9) Forced seclusion (#9) Isolation due to public reaction (#3)	Retreating to isolation	Social Concerns
The black sheep of the family (#8) Lack of family support (#6) Religious bias (#7) Forced and disgusting marriage (#3)	Stressful family conditions	
Non-acceptance of sexual identity by others (#1) Ambiguity in identity for others (#9) Others’ surprise for being a woman or a man (#9) Being judged (#4)	Lack of public recognition	

### A. Living in agony

One of the challenges pointed out by the participants in this study was living in agony due to annoying physical characteristics and mental suffering (Table 2).

#### Annoying physical characteristics

The participants stated that they had to live in agony due to annoying physical features. Thus, they suffered from female appearance, hid and disliked their feminine characteristics, and hated their feminine appearance. Accordingly, one of the participants stated:

“*I usually try not to look at myself in the mirror from the neck down. It bothers me to see this feminine look. I try not to be seen in any way. Sometimes I have to wear a chador*” (Participant #2).

Another participant narrated her childhood memories:

“*I remember the first day of primary school when I was sent to a girls’ school. I was crying about why my parents didn’t send me to a school for boys. Even my mother told me when I was 4 years old, I cried a lot when they asked me to wear female dresses and earrings while going to a relative’s wedding and I told them I was a boy and why should I wear them*” (Participant #4).

#### Mental suffering

The participants stated that they had a lot of mental suffering because of distress, unforgivable sin, endless abuse, guilt, living in a cage, extreme humiliation, and being the black sheep of the family. One of the participants stated:

“*Despite my religious beliefs, I had a lot of mental suffering that turned my life into a real hell. Even if God said you shouldn’t do this, I couldn’t accept it*” (Participant #6).

Another participant described the deprivation from gender identity:

“It *bothers me a lot that I cannot be a perfect man even by undergoing gender reassignment surgery. I have told God many times why, despite all the efforts I have made to be a good person and not commit any sin, I have to suffer a lot for being deprived of the simplest blessing, which is having a body that matches my identity*” (Participant #1).

#### Disturbing sexual changes

The participants reported concerns about their sexual changes including concerns about impotence, their appearance after gender reassignment surgery, marriage after the surgery, their sexual partners, and fertility after the surgery. One of the participants said:

“*One of my concerns is that after surgery and hormone therapy, I will not have the feminine look and physique I desire*” (Participant #3).

Another participant stated: “*I think a lot about my marriage after the surgery. I wonder if there will be someone who will accept me with these conditions*” (Participant #4).

#### Concerns about public reaction

The participants reported that they were afraid of other people’s reaction to their new sexual identity and they were worried about the parents’ reaction, the family’s behavior, not being accepted after the surgery, people’s curiosity about their previous sexual identity, and the lack of public recognition of their new identity. One of the participants stated: “*I am worried that I will not be recognized as male by the people around me even after the gender reassignment surgery*” (Participant #1).

Another participant was worried that others would find out about his former sexual identity: “*I am worried that after the surgery, people will find out about my past and my previous gender identity*” (Participant #8).

### B. Confusion

One of the challenges reported by the people with gender dysphoria was confusion that led to their helplessness, surrender, and the final solution (Table 2):

#### Helplessness

The participants stated that they felt helpless due to the lack of family support in choosing the type of clothing, confusion about their sexual identity, other people’s confusion about their identity, and complaints about God. One of the participants stated:

“*Once my sister and I went to a bank to open a bank account. We were wearing male clothes. The bank clerk refused to open a bank account and said that our identity documents belong to two girls, but you are boys*” (Participant #1).

Another participant stated:

“*My parents were confused from the very beginning. They realized that I was different from the others, but they didn’t know exactly that I had gender dysphoria. They only knew that I had girlish behaviors. Even my mother told me that when I was 4, I used to play with the girl next door and I preferred to play with dolls*” (Participant #5).

A third participant reported:

“*At first, I didn’t know exactly what condition I was suffering from and I only knew that I was different from other children of my age and this was a secret that I had to keep with me and I really felt helpless*” (Participant #6).

#### Surrender

The participants reported that they had to surrender to the challenges faced by them such as wearing clothes according to local norms, other people’s surprise at their identity, family conflict, and futile attempts to keep their identity. One of the participants said:

“*I have to wear girl’s clothes outdoor in public places; otherwise, I will face other people’s surprise or their judgmental behavior*” (Participant #4).

Another participant stated:

“*Although I had a boyish appearance at school, my friends and school staff said that I behaved like a girl, and I was teased many times by my classmates and teachers. At first, it was very painful for me, but I got used to it little by little*” (Participant #5).

#### The final solution

The participants stated that they had to make a final decision to overcome their challenges such as insistence, preparing for independence or being rejected after the gender reassignment surgery, leaving school due to clothing not fitting their gender, committing suicide when feeling helpless, migration, and deciding to have a specific gender identity. One of the participants stated:

“*If I conclude that there is no way out of this situation, I will think of committing suicide*” (Participant #4).

However, another participant stated:

“*Finally, I will do a gender reassignment surgery, but probably I will be rejected by my family, or my current partner will break her relationship with me. In any case, true identity is important to me*” (Participant #3).

### C. Social concerns

The participants in this study talked about their social concerns and stated that retreating to isolation, stressful family conditions, and lack of public recognition were their main social concerns and challenges (Table 2).

#### Retreating to isolation

The participants reported that they preferred to retreat to isolation due to their annoying clothing, feeling safe in isolation, fear of the disclosure of their true identity, low self-confidence, and disillusionment with their current identity. One of the participants stated:

“*We had a female classmate with a lot of extra hair on her body due to hormonal problems. The students rumored that she was bisexual. This rumor spread quickly in the university, and unfortunately, it had a bad psychological effect on that girl so she had to continue her studies at another university. This caused a lot of anxiety for me and I was very afraid of the students finding out about my problem. Thus, I had to not communicate with anyone*” (Participant #8).

Another participant stated:

“*I don’t go out much, but sometimes when I go to a coffee shop or restaurant with my partner, especially in men’s clothes, people look at us with surprise, and some even point at us mockingly and say that we are gay. Things get worse and they make fun of me when I start talking. Well, I don’t come out anymore*” (Participant #3).

#### Stressful family conditions

The participants reported stressful family conditions as one of their social concerns caused by the tense psychological atmosphere in the family, forced and disgusting marriage, the sense of being the black sheep of the family, the persuasion of the parents, and religious bias. One of the participants stated:

“*On the street and in places where I feel that someone might recognize me, I wear girl’s clothes or appear less frequently because no one except my family members knows about this. The reputation of my family is very important to me and I am afraid of people’s reaction*” (Participant #4).

Another participant stated:

“*My father forced me to get engaged to a girl. We didn’t have any kind of emotional relationship, and even when she sat next to me, she gave me a disgusting feeling. There was a lot of tension in the family because of my resistance to getting married, but in the end, I decided to break up with her*” (Participant #3).

#### Lack of public recognition

Another social concern of the participants was the lack of public recognition including non-acceptance of appearance characteristics, futile attempts to convince others, public unawareness, non-acceptance of gender identity by others, worry about police threats, people surprise, obstacles to finding a job, public judgment, and interrogation in public and governmental departments and offices. A participant stated:

“*When I went to the court to get an introduction letter to the forensic medicine department, the judge reacted badly and said that there was no such thing, as a person can be either a man or a woman, and all these were my illusions. Upon hearing this answer, I had a terrible feeling*” (Participant #9).

Another participant reported:

“*I was busy painting a building. I heard from one of my friends that somebody told the owner of the house that the guy who was painting the house was bisexual and he wants this money for the gender reassignment surgery. He had convinced the owner that he would commit a great sin if he allowed me to paint the house and he would be disgraced. Thus, the owner dismissed me*” (Participant #6).

Another participant stated:

“*I can never go to public places with peace of mind because I hate other people’s judgments about me. For example, they say I’m bisexual or how ugly it is for a girl to act like a boy or other things that they may say about me*” (Participant #4).

## Discussion

This study sought to examine the physical, psychological, and social challenges faced by people with gender dysphoria. The data analysis showed that living in agony, confusion, and social concerns were the main challenges faced by people with gender dysphoria. The participants stated that they were living in agony due to their annoying physical characteristics and they hated and tried to hide feminine features. In their study from 2007 to 2012 in Amsterdam, van de Grift et al. showed that having a physical appearance inconsistent with gender identity may lead to more difficult psychological adaptation and exposure to discrimination and stigma [2]. Moreover, in their study in England, Coleman-Smith et al. (2020) showed that all the participants reported distress and suffering as a result of the mismatch between their gender identity and the gender they were born with [14]. A study by Campbell et al. (2018) in Africa showed that many people with gender dysphoria have a strong desire to have the opposite gender due to their suffering caused by their different physical characteristics [15]. It seems that the participants’ suffering due to their physical characteristics is caused by the mismatch between their true gender identity and their biological gender, and this mismatch can be the source of a lot of psychological harm in these people.

Another challenge reported by the participants with gender dysphoria was their concerns about sexual changes due to the development of secondary sexual characteristics, as well as functional impairment and the inability to have sex after the gender reassignment surgery, and concerns about the public reaction after realizing their conditions and their previous gender identity. In their study in Turkey, Yıldızhan et al. (2018) showed people who had performed gender reassignment surgery had reduced anxiety related to gender discrimination, but increased anxiety related to disclosure of their past compared to the group who did not perform this surgery [4]. Another study by Holt et al. (2016) in England showed that the level of anxiety and worry in people with gender dysphoria increases when reaching puberty, so a significant percentage of these people experienced suicidal thoughts, low mood, and depression [16]. Another study conducted by Ross and Need (1989) in the United States showed that people with gender dysphoria have concerns about sex reassignment, including erectile dysfunction, lack of social support, family reactions, urinary incontinence, and the need for more surgeries [17]. Following these findings, people with gender dysphoria develop concerns about the occurrence of changes caused by secondary sexual characteristics and the intensification of the conflict between their true sexual identity and their physical appearance. Besides, the sexual changes developed after gender reassignment may not be efficient in creating a proper sexual relationship. In addition, public unawareness of the conditions of these people and the conflict between their bodies and gender identity before the surgery can make other people mistreat people with gender dysphoria. However, after the surgery, people’s awareness of their past conditions and the lack of public recognition may create concerns in these people.

The patients with gender dysphoria in this study also reported that sometimes they felt confused and helpless and thus they decided to commit suicide. Some of the participants stated that if for any reason they could not undergo gender reassignment surgery, they would inevitably surrender to it and would live in distress caused by the conflict between their true gender identity and their body for the rest of their lives. A study by Ghazanfari et al. (2018) in Iran showed that due to the stigma caused by gender dysphoria, a large number of people with this condition never go to a psychiatrist, some emigrate and some spend the rest of their lives in distress, and confusion caused by the conflict between body and soul [18]. Another study by Mantashloo et al. (2019) in Iran showed that psychological pain, feelings of helplessness, withdrawal from life events, isolation, and suppression of emotions are more common in people with gender dysphoria [19]. In their study in Canada, Aitken et al. (2016) found that one of the possible reasons for the presence of suicidal thoughts and self-harm behaviors in people with gender dysphoria is that gender dysphoria itself inherently causes distress and confusion in these people [20]. Moreover, a study by Peterson et al. (2017) in the United States showed that the prevalence of suicide attempts and self-harm was higher in people who tended to have a male gender than those who tended to have a female gender [8]. It seems that a feeling of helplessness and confusion in these people is caused by psychological distress due to gender dysphoria that they have experienced. Besides, one of the reasons why these people surrender to the special conditions they experience and do not perform gender reassignment surgery can be the stigma caused by gender dysphoria in Iran. Under the influence of cultural and religious norms, people consider gender reassignment surgery as a sin, making the performance of this surgery more difficult for people with gender dysphoria, and this can intensify the feeling of helplessness and confusion in these people and force them to surrender to the status quo.

The participants in this study talked about their social concerns and stated that retreating to isolation, stressful family conditions, and lack of public recognition were their main social concerns and challenges. However, Wong and Drake (2017) evaluated the experiences of parents who allowed their children with GD to change social roles at an early age in Canada and showed that these children had better social relations and more effective interactions with their parents [21]. Javaherian and Koochakian (2006) showed that although gender reassignment surgery is allowed in Iran’s legal system, people with gender dysphoria face many social and cultural problems in private and public areas [22]. Furthermore, in their study in Iran, Rahimi Ahmadabadi et al. (2016) showed that people with gender dysphoria scored higher than the control group in terms of stress, anxiety, depression, and gender role disorder. They also found that raising parents’ awareness cannot solve the problems associated with gender dysphoria because it has cultural, social, and reputational aspects [23]. In another study in Iran, Heidari et al. (2020) indicated that the reaction of the family members of people with gender dysphoria to their situation follows a wide spectrum and families do their best to avoid shame and embarrassment in the community [24]. Previous studies have also reported that people with gender dysphoria may be rejected by their families, especially after they reveal their true gender identity. They may also lose support from their families and friends. These studies show that people with gender dysphoria who sought treatment did not have the desire to find new friends until they completed gender reassignment procedures, and all these cases lead to the isolation of these people [25, 26].

It seems that most of the challenges faced by people with gender dysphoria are caused by unawareness of their conditions by the family and the public, which in turn is caused by the failure of related organizations and experts to provide adequate information about the conditions of these people. Thus, psychological and culture-based interventions may be effective in solving these challenges.

One of the limitations of the present study was the unavailability of people with gender dysphoria. Thus, to collect adequate data, additional interviews were conducted with the participants to address all aspects of the issue in question.

## Conclusion

The findings of the present study show the numerous personal and social challenges of people suffering from gender dysphoria, which appeared in the classes of “living in agony”, “confusion” and “social concerns”. The findings indicate that the challenges of these people are caused by the lack of understanding of their conditions by the family and society, and it is a sign of the lack of awareness of families and communities about their conditions and poor information from the responsible institutions, so the results of this study can be By recognizing the challenges, it can be the solution to the problems of these people. It is suggested to conduct a qualitative study on the experiences of these people’s families.

## Data Availability

The datasets generated and/or analyzed during the current study are not publicly available to protect the privacy of study participants but are available from the corresponding author upon reasonable request.
